# Mealtime Physiological Responses in Individuals With Eating Disorders and Healthy Controls

**DOI:** 10.1002/erv.70022

**Published:** 2025-08-02

**Authors:** Emma De Schuyteneer, Robin Quagebeur, Femke de Gooijer, Annelies Goris, Neide Simões‐Capela, Alex van Kraaij, Elske Vrieze

**Affiliations:** ^1^ Mind‐body Research Biomedical Sciences Group KU Leuven Leuven Belgium; ^2^ Leuven Brain Institute KU Leuven Leuven Belgium; ^3^ OnePlanet Research Center Wageningen the Netherlands; ^4^ Division of Human Nutrition and Health Department of Agrotechnology and Food Sciences Wageningen University and Research Wageningen the Netherlands; ^5^ Department of Electrical Engineering (ESAT) KU Leuven Heverlee Belgium; ^6^ IMEC Heverlee Belgium

**Keywords:** anorexia nervosa, bulimia nervosa, food, physiology, stress

## Abstract

**Objective:**

Mealtimes are highly distressing for individuals with eating disorders (ED), potentially reinforcing disordered eating and complicating recovery. Yet, physiological responses during meals remain understudied. This study explores autonomic nervous system responses during meals in individuals with ED and healthy controls (HC).

**Methods:**

Three studies assessed heart rate (HR), heart rate variability (HRV), skin conductance response (SCR), and skin temperature (ST) around lunchtime. Study 1 included 47 hospitalized adult women with ED (26 anorexia nervosa (AN), 8 atypical AN, 13 bulimia nervosa (BN)). Studies 2 and 3 involved 47 and 58 HC in daily life.

**Results:**

Patients reported elevated subjective stress but showed no expected physiological responses in HR. Instead, HR decreased during meals for all patients and increased after in those with BN. No significant changes were observed in HRV or SCR. No ST changes were observed in AN, while BN showed the expected pre‐lunch decrease and post‐lunch increase. In HC, HR and SCR rose pre‐meal, HRV decreased, and ST increased during meals.

**Discussion:**

These findings suggest a mismatch between subjective and physiological stress in ED. Chronic stress or undernutrition may alter autonomic reactivity, although not directly assessed. Future research should investigate how these factors shape physiological stress responses.

## Highlights

- Mealtimes are associated with higher levels of psychological stress in individuals with anorexia nervosa and bulimia nervosa and can be considered as an eating disorder‐specific stressor.
- Despite reporting high stress, physiological stress markers (heart rate variability, skin conductance, and skin temperature) do not clearly indicate a stress response in these individuals.
- Autonomic nervous system changes observed during lunch in healthy controls are not related to subjective stress.

**Abbreviations**
ACC: Acceleration
AN: Anorexia nervosa
ANS: Autonomic nervous system
BBI: Beat‐to‐Beat Interval
BN: Bulimia nervosa
DEBQ: Dutch Eating Behavior Questionnaire
ECG: Electrocardiogram
ED: Eating disorders
EDI‐3: Eating Disorder Inventory‐3
HC: Healthy controls
HPA axis: Hypothalamus–pituitary–adrenal axis
HR: Heart rate
HRV: Heart rate variability
JITAI: Just‐in time adaptive intervention
PPG: Photoplethysmography
PSS‐10: Perceived Stress Scale‐10
RMSSD: Root mean square of successive RR differences
SC: Skin conductance
SCR: Skin conductance response peaks per minute
SDNN: Standard deviation of the NN intervals
SNS: Sympathetic nervous system
SQI: Signal quality indicator
ST: Skin temperature
VAS: Visual Analogue Scale

## 1 Introduction

Anorexia nervosa (AN) and bulimia nervosa (BN) are eating disorders (ED) characterized by disordered eating behaviours, which include restrictive food intake, binge eating and purging, accompanied by a preoccupation with body shape and weight, as well as maladaptive thoughts and beliefs about food (APA 2013). Stress plays an important role in the development and progression of AN and BN, influencing both psychological and biological processes. The body's stress response is primarily mediated by two systems: the hypothalamus–pituitary–adrenal axis (HPA axis) and the sympathetic nervous system (SNS) (Marques et al. 2010). As the current study used data collected by wearable devices that captured only autonomic nervous system responses, we focused specifically on SNS activity.

Evidence from individual studies suggests that individuals with ED often exhibit autonomic nervous system (ANS) dysregulation. Specifically, studies indicate that individuals with AN, at rest, show lower heart rate (HR) and skin conductance (SC) and higher heart rate variability (HRV) compared to healthy controls (HC) (Jenkins et al. 2021; Ralph‐Nearman et al. 2024). The differences observed in these studies appear largely related to the somatic consequences of being underweight but do not fully rule out the possibility of disorder‐specific alterations. In individuals with BN, HRV tends to be higher, while SC and skin temperature (ST) are lower compared to HC (Ralph‐Nearman et al. 2024). However, understanding stress reactivity in ED is more complex due to several factors, including variability across diagnostic groups, differences between laboratory and naturalistic settings, inconsistencies in physiological findings, and the difficulty of disentangling state‐related effects from potential trait‐like alterations. Most existing research has focused on emotional and autonomic responses to non‐meal‐related stressors, such as interpersonal stress, typically studied in controlled laboratory settings. A systematic review reported that individuals with ED often exhibit heightened emotional responses to interpersonal stress, coupled with blunted physiological reactivity, suggesting a disconnect between subjective experience and biological stress markers (Monteleone et al. 2018). In contrast, the current study investigates stress reactivity during mealtimes, a real‐life, ED‐specific stressor, in a naturalistic clinical setting, providing ecologically valid insights into stress responses in ED.

While interpersonal stress has been a primary focus so far in research, it might be worthwhile to examine ED‐related stressors more closely. For individuals with ED, mealtimes and the postprandial period are associated with heightened levels of negative emotions and maladaptive cognitions (Levinson et al. 2018; Long et al. 2012), as evidenced by laboratory studies showing that exposure to food images increases negative emotions (Foroughi et al. 2020). Moreover, elevated levels of distress and anxiety have also been observed during hospital dinners (Accurso et al. 2023) and controlled test meals in laboratory settings (Buree et al. 1990). Additionally, pre‐meal anxiety has been correlated with a reduction in caloric intake in patients with ED (Lloyd et al. 2021; Steinglass et al. 2010). Unfortunately, research has predominantly focused on subjective stress experiences around mealtime, with fewer studies exploring physiological stress responses. The limited research suggests inconsistent findings, with some studies reporting heightened autonomic responses to food cues and others indicating blunted reactions (Christensen et al. 2020; Gordon et al. 2001; Gorini et al. 2010; Soussignan et al. 2011). Only one study investigated autonomic responses to an actual meal, finding that HR peaked during an actual meal and declined afterward, while SC peaked at the onset of eating in both individuals with ED and HC (Buree et al. 1990).

To address this gap, this paper presents three studies investigating physiological responses to actual meals in individuals with ED and HC. We use wearable sensors, which offer a non‐invasive, passive method for recording physiological measures, to capture the body's response in a naturalistic setting (Presseller et al. 2022). Study 1 examines physiological and subjective stress responses to a standardised lunch in hospitalized individuals with AN and BN. The study seeks to determine whether mealtimes elicit specific patterns of autonomic nervous system activation that differ from those observed in HC, and whether these patterns align with self‐reported stress levels. We hypothesized an anticipatory increase in subjective stress before lunch, accompanied by physiological signs of parasympathetic withdrawal and sympathetic activation, reflected in increased HR and SC and decreased HRV and ST. After the meal, we expect HR and SC levels to decrease and HRV and ST to increase (Mccorry 2007; Vinkers et al. 2013). Additionally, we will explore whether physiological variables remain stable, increase, or decrease during the meal itself. Studies 2 and 3 aim to examine physiological responses to mealtime in HC within a naturalistic office setting, providing a baseline for non‐stressful mealtime reactions. In HC, we anticipated minimal or normative physiological changes throughout the mealtime period, without pronounced stress responses.

## 2 Study 1: Physiological Responses During Lunch in Individuals With ED

### 2.1 Materials and Methods

#### 2.1.1 Participants

This study was conducted from February 2018 to September 2019 at the Mind‐Body Unit of the University Psychiatric Center, KU Leuven, in Belgium. A detailed study protocol is available in Simões‐Capela et al. (2019). While the original study encompassed a broader sample, this manuscript focuses on a specific subset of 47 adult women (age 18–50 years, *M* = 26.1, SD = 7.4), of which 26 had a diagnosis of AN, 8 had atypical AN and 13 had BN, based on DSM‐5 criteria (APA 2013). All patients received treatment for their ED at the Mind‐Body Unit, including meals at the hospital for at least one day weekly. Details on the broader dataset from the original study, its aims, and the rationale for subsample selection are provided in the Supporting Information S1.

The protocol was approved by the Medical Ethical Committee of UZ Leuven and all participants signed informed consent. [Corrections added on 11 November 2025, after first online publication: Redacted text in Section 2.1.1 have been corrected to ‘Mind‐Body Unit’, ‘KU Leuven’, ‘Belgium’, ‘Simões‐Capela et al. (2019)’, and ‘UZ Leuven’. Details for the previously redacted in‐text citation have been included in the References.]

#### 2.1.2 Procedure

On study mornings, participants were fitted with wearables and completed baseline questionnaires. Then, participants continued with their normal day, while physiology was continuously measured until 5 p.m. Additionally, participants completed diary entries every hour to report their subjective stress levels. Meals took place from 12:15 p.m. to 12:45 p.m. in the designated eating room on the ED treatment ward, under consistent environmental conditions across days. While meals were not identical each day, they were broadly comparable in caloric content and composition, as they adhered to the structured menu developed by the ward's dietitian. Participants ate at the same table with other patients undergoing ED treatment under clinical staff supervision. In line with the unit's protocol, patients remained in the dining area for 30 min after the meal, minimizing the opportunity for immediate compensatory behaviours.

### 2.2 Assessment

#### 2.2.1 Wearable Sensors

Participants wore two types of wearable sensors. The Chillband (IMEC vzw, Belgium) is a wrist‐worn device, and participants wore one on each wrist. It measures skin temperature (ST; 1 Hz), skin conductance (SC; 256 Hz) and acceleration (ACC; 32 Hz). Secondly, participants wore an electrocardiogram chest patch (Biotelemetry, Denmark), measuring electrocardiogram variables (ECG; 256 Hz) and acceleration (32 Hz). Although these devices are not standard clinically validated equipment, they have been specifically developed for research purposes. The preprocessing steps are described in the Supporting Information S1.

#### 2.2.2 Questionnaires

Participants completed the following self‐report questionnaires: the Eating Disorder Inventory‐3 (EDI‐3; Garner 2004), measuring eating disorder psychopathology; the Dutch Eating Behavior Questionnaire (DEBQ; Van Strien et al. 1986), evaluating general eating styles and behaviours; and the Perceived Stress Scale‐10 (PSS‐10; Cohen et al. 1983), assessing subjective stress levels over the past month.

#### 2.2.3 Self‐Report Diary

Participants answered the following question: ‘How stressed are you feeling right now?’ on a visual analogue scale (VAS) from ‘not stressed at all’ to ‘extremely stressed’ at three different time points, namely 45 min before lunch, in the middle of lunch, and 45 min after lunch.

#### 2.2.4 Statistical Analysis

All statistical analyses were conducted using R Studio (R Core Team 2021). The statistical models testing our main hypotheses were registered on the Open Science Framework (https://osf.io/4vewy). In this manuscript, we report the analyses with an interaction with Diagnosis (AN or BN), the models without are described in the Supporting Information S1 (Table S2, Table S4).

Group comparisons were performed using either an unpaired *t*‐test for normally distributed data or a Wilcoxon rank‐sum test for non‐normally distributed data.

To test the hypothesis regarding physiological variables, we used different piecewise linear mixed models, using the nlme (Pinheiro and Bates 2023) and lspline (Bojanowski 2017) packages in R. These models allow for distinct linear relationships between the predictors and the outcome variable within specific data intervals, with our physiological variables as the outcomes and the variable Time (continuous), Diagnosis (0 = AN or atypical AN, 1 = BN) and the interaction between Time and Diagnosis as predictors. We added two breakpoints to the model, at the start and the end of lunch. The standard deviation of acceleration (measure for movement, not mentioned in preregistration) and BMI were added as covariates. The variable SCR was log‐transformed to better fit a normal distribution. All models included a random intercept, and the rest of the random part was produced bottom‐up using likelihood ratio tests. The statistical analyses were done on the final models including a random intercept for each participant and a random slope for Time. We did not include quadratic Time variables, as preregistered, because of convergency issues. Moreover, we were unable to fit a model using tonic skin conductance level as the outcome, as initially preregistered, because this variable was only available in a previously processed format, which did not allow accurate analysis. Moreover, we tested the model without BMI as a covariate due to multicollinearity between BMI and Diagnosis. The results were similar to those obtained with BMI included in the model.

Due to technical issues, two participants did not have chest patch data, and three did not have Chillband data. The percentage of missingness is 46.61% for the chest patch data and 41.09% for the Chillband.

As a manipulation check, we employed a linear mixed model with Subjective Stress as the outcome variable and the categorical variable Time (before, after, reference category = during), Diagnosis and their interaction as predictors. We included a random intercept for each participant. Two values were missing. While most assumptions of the model were satisfied, the assumption of homogeneity seemed to be violated. Therefore, we fitted a robust linear mixed model as a sensitivity analysis, and the results remained consistent with the initial model.

## 3 Results

### 3.1 Clinical and Demographic Characteristics

Table 1 summarizes the clinical characteristics. Additionally, Table S1 (Supporting Information S1) provides a sub‐analysis that compares various clinical variables between the AN and BN participants.

**TABLE 1:** Descriptive statistics of demographic and clinical characteristics of the included studies.

Descriptives	Study 1	Study 2	Study 3	Test statistic
	*M (*SD*); range*	*M (*SD*); range*	*M (*SD*); range*	
*N*	47	27	57	/
Age, years	26.1 (7.4); 18.0–50.0	29.3 (4.5); 22.0–39.00	27.1 (6.1); 18.0–48.0	H (2) = 9.04 * Study 1 vs. Study 2 * Study 2 vs. Study 3 *
BMI	18.5 (3.7); 11.5–28.6	/	22.5 (3.1); 14.8–30.8	*t* (90.077) = −5.80 ***
Days since treatment start	36.4 (29.4); 3–105	/	/	/
Gender				
Female	47	19	31	/
Male	0	8	25	/
Non‐binary	0	0	1	/
Questionnaires				
EDI total score	175.0 (45.6); 85–285	/	/	/
PSS Score	27.9 (4.7); 14–37	16.0 (5.3); 5–25	/	*W* = 1221 ***
DEBQ restrained eating	4.1 (0.8); 2.0–5.0	1.8 (0.8); 0.1–3.1	2.1 (0.5); 1.1–3.1	H (2) = 79.36 *** Study 1 vs. Study 2 *** Study 1 vs. Study 3 ***
DEBQ external eating	2.6 (0.9); 1.0–4.4	2.9 (0.8); 0.6–4.5	3.4 (0.5); 2.4–4.6	F (2,57) = 15.69 *** Study 3 vs. Study 1 *** Study 3 vs. Study 2 *
DEBQ emotional eating	2.3 (1.2); 1.0–4.8	1.9 (1.2); 0.0–3.8	2.6 (0.7); 1.1–4.2	F (2,58) = 4.07 * Study 2 vs. Study 3 *

*Note:* The results of the post hoc comparisons are displayed with * if *p* < 0.05, ** if *p* < 0.01 and *** if *p* < 0.001.

### 3.2 Physiological Variables Around Lunch

#### 3.2.1 Heart Rate

**FIGURE 1:**
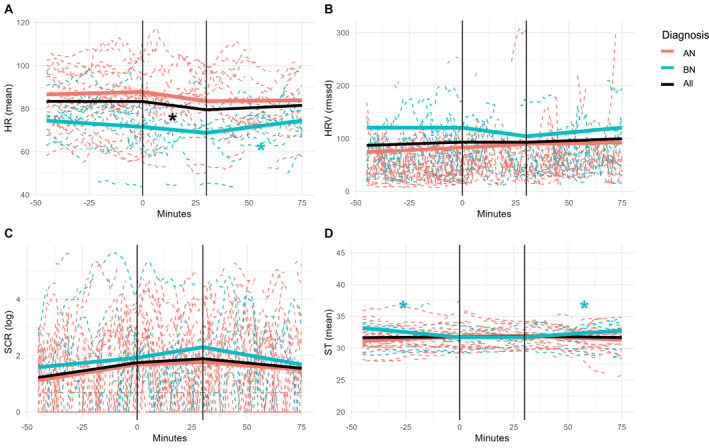
Physiological variables over time relative to meal. The vertical lines represent the start and the end of the lunch. The horizontal dashed lines are the individuals' observed values, and the solid lines are the predicted models. The red lines show the results for the individuals with AN, the blue lines for the individuals with BN, and the black line represents the entire sample. Panel A shows heart rate (HR), panel B shows heart rate variability (HRV; rmssd), panel C displays skin conductance response (SCR; log‐transformed), and panel D presents skin temperature (ST).

Contrary to our hypotheses, HR did not significantly increase before lunch across groups (*β* = 0.03, *t (2834)* = 0.93, *p* = 0.350) (Figure 1A and Supporting Information S1: Table S5). During lunch, HR significantly decreased in the total sample (*β* = −0.14, *t (2834)* = −3.76, *p* = < 0.001). After lunch, individuals with AN showed no significant change in HR (*β* = 0.01, *t (2834)* = 0.29, *p* = 0.775), whereas individuals with BN showed a significant HR increase (*β* = 0.12, *t (2831)* = 1.98, *p* = 0.048). This significant Time × Diagnosis interaction suggests group‐specific responses over time.

We found no main effect of BMI (*β* = 0.44, *t (43)* = 0.70, *p* = 0.488). There was a significant main effect of ACC (*β* = 110.79, *t (2831)* = 12.24, *p* = < 0.001), and a significant main effect of Diagnosis, with lower overall HR in individuals with BN compared to AN (*β* = −16.21, *t (42)* = −3.01, *p* = 0.004).

#### 3.2.2 Heart Rate Variability

Contrary to our hypotheses, we did not find a significant decrease in RMSSD before lunch across groups (*β* = 0.20, *t (2834)* = 1.30, *p* = 0.192) nor a significant increase after (*β* = 0.05, *t (2834)* = 0.22, *p* = 0.829) (Figure 1B, Supporting Information S1: Table S5). In addition, we did not find a main effect of Time during lunch (*β* = 0.25, *t (2834)* = 0.97, *p* = 0.330). We found no significant interactions between Time and Diagnosis (all interaction statistics reported in Supporting Information S1: Table S5). We found no main effect of BMI (*β* = −3.16, *t (43)* = −1.54, *p* = 0.131). We observed a significant main effect of ACC (*β* = 1070.76, *t (2831)* = 15.10, *p* = < 0.001).

#### 3.2.3 Skin Conductance Response

Contrary to our hypotheses, we did not find a significant increase in the log‐transformed SCR before lunch across groups (*β* = 0.01, *t (2863)* = 1.81, *p* = 0.070) nor a significant decrease after lunch (*β* = −0.01, *t (2863)* = −0.89 *p* = 0.374) (Figure 1C, Supporting Information S1: Table S5). There was no significant main effect of Time during lunch across groups (*β* = 0.00, *t (2863)* = 0.39, *p* = 0.700). We found no significant interactions between Time and Diagnosis (Supporting Information S1: Table S5). There was no main effect of BMI (*β* = 0.01, *t (39)* = 0.10, *p* = 0.923) or ACC (*β* = −0.30, *t (2863)* = −0.71, *p* = 0.475).

#### 3.2.4 Skin Temperature

Contrary to our hypotheses, we did not find a significant decrease in ST before lunch in individuals with AN (*β* = 0.01, *t* (2863) = 1.31, *p* = 0.191), nor a significant increase after lunch (*β* = −0.01, *t* (2863) = −1.87, *p* = 0.062) in this group (Figure 1D, Supporting Information S1: Table S5). However, significant Time × Diagnosis interactions emerged for both the pre‐ and post‐lunch phases. Specifically, ST significantly decreased before lunch in individuals with BN (interaction: *β* = −0.05, *t* (2860) = −2.10, *p* = 0.035) and increased after lunch (interaction: *β* = 0.04, *t* (2860) = 2.66, *p* = 0.008) in this group. No significant main effect of time during lunch was observed (*β* = 0.00, *t (2863)* = 0.43, *p* = 0.667), nor were there main effects of BMI (*β* = 0.02, *t (39)* = 0.20, *p* = 0.846) or ACC (*β* = 0.08, *t (2863)* = 0.41, *p* = 0.685).

#### 3.2.5 Subjective Stress Around Lunch

**FIGURE 2:**
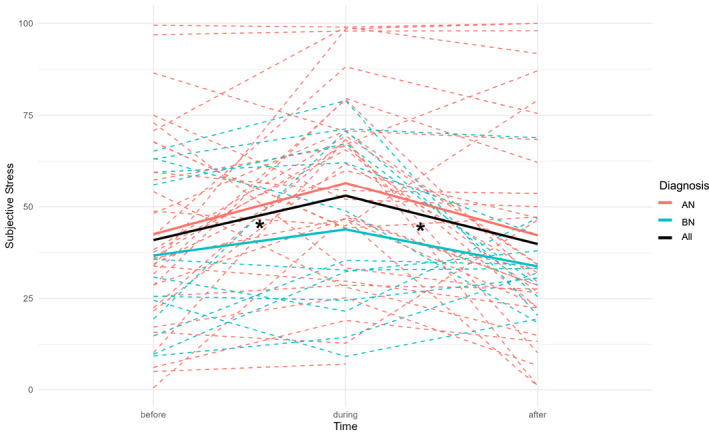
Subjective stress before, during and after lunch in individuals with eating disorders. The dashed lines represent the observed values for each individual. The black line is the predicted model of the total group, the red line is the predicted model of the AN group and the blue line is the predicted model of the BN group.

While no clear physiological stress patterns emerged around the meal, subjective stress ratings showed a clear temporal pattern. As hypothesized, subjective stress significantly increased during lunch across groups and was lower both before (*β* = −14.00, *t (134)* = −3.25, *p* = < 0.001) and after lunch (*β* = −14.32, *t* (134) = −3.32, *p* < 0.001) when compared to the lunch period (Figure 2, Supporting Information S1: Table S3). There was no significant main effect of Diagnosis (*β* = −12.73, *t (*131) = −1.57, *p* = 0.119) or interactions between Time and Diagnosis (Supporting Information S1: Table S5).

## 4 Study 2 and Study 3: Physiological Responses During Lunch in HC

### 4.1 Method

#### 4.1.1 Participants

For Study 2, we used data previously collected in 2022 (de Gooijer et al. 2023) and for Study 3 in 2023 (van Kraaij et al., in preparation). Study 2 is a non‐randomized, non‐blinded study investigating ANS with wearables during meals and snack time for 5 days (de Gooijer et al. 2023), and Study 3 is a randomised, single‐blinded study investigating a just‐in‐time adaptive intervention (JITAI) with wearables measuring ANS on snacking behaviour for 2 weeks. Respectively, 47 and 58 adults (age 18–49 years) without psychiatric illnesses or dietary restrictions were included in the studies. Both studies were deemed exempt from review for ethical approval according to the Dutch Medical Research Involving Human Subjects Act (WMO) by the medical‐ethical committee of the Maxima Medical Center in Veldhoven, Netherlands.

A detailed study protocol is available in (de Gooijer et al. 2023). This manuscript focuses on the physiological data surrounding lunch in both studies. For Study 3, only data from the baseline week, without JITAI, was analysed. 27 Participants in Study 1 and 58 participants in Study 2 reported the moment they had lunch (between 1 and 5 times). We analysed physiological data surrounding the participants' self‐reported lunch moments from 45 min before lunch to 75 min after the reported start of their meal (see Supporting Information S1 for further details). [Corrections added on 11 November 2025, after first online publication: Redacted text in Section 4.1.1 have been corrected to ‘(van Kraaij et al., in preparation)’, ‘(de Gooijer et al. 2023)’, ‘Dutch Medical Research Involving Human Subjects Act (WMO)’, and ‘the Maxima Medical Center in Veldhoven, Netherlands’.]

### 4.2 Assessment

#### 4.2.1 Wearable Sensors

In Study 2, participants wore the Garmin Vivosmart 4 measuring photoplethysmography (PPG) data to derive *β* heart rate (HR) and the Chill+ (IMEC vzw, Belgium) measuring skin temperature (1 Hz), skin conductance response peaks per minute (64 Hz) and acceleration (32 Hz). In Study 3, the Garmin Vivosmart 5 smartwatch was worn for up to ten (work)days within a 14‐day period, deriving *β* heart rate using PPG data. Garmin data was accessed via the GarminHealth SDK. The preprocessing steps are described in the Supporting Information S1.

#### 4.2.2 Questionnaires

Participants in both Study 2 and Study 3 completed the Dutch Eating Behavior Questionnaire (DEBQ; Van Strien et al. 1986 and the Perceived Stress Scale‐10 (PSS‐10; Cohen et al. 1983). Additionally, meal timing was reported using the Traqq food recall application (Lucassen et al. 2021).

#### 4.2.3 Self‐Report Diary

Throughout the day, participants received prompts via another application to report their subjective stress levels at various intervals using a Likert scale from relaxed (0) to stressed (100).

### 4.3 Statistical Analysis

For subjective stress scores reported during lunchtime, when multiple scores were available for a given participant, we calculated the average of these scores to create a single value for each participant. These averaged stress scores were then statistically summarized at the group level.

To explore the trajectories of physiological variables around lunch, we used piecewise linear mixed models with the physiological variables as outcomes and the variable Time (continuous) as predictor. We added two breakpoints to the model, at the reported start of the lunch and after 30 min. For Study 2, gender and the standard deviation of acceleration (as a measure of movement) were added as covariates. For Study 3, gender, BMI and the step count (as a measure of movement) were added as covariates. One individual identified as non‐binary and was excluded from the model with Gender as a covariate due to their single representation. All models included a random intercept, and the rest of the random part was produced bottom‐up using likelihood ratio tests. The statistical analyses were done on the final models including a random intercept for each participant, a nested variable Meal ID within each participant and a random slope for Time. Except for SCR, where there was a better model fit without a random slope for Time. All assumptions were met.

Missing data in Study 2 was 16.69% for HR, 23.77% for SCR, and 4.90% for ST, while in Study 3, it was 10.43% for HR and 7.20% for HRV due to low‐quality segments.

## 5 Results

### 5.1 Demographic Characteristics

Table 1 summarizes demographics and clinical characteristics.

### 5.2 Physiological Variables Around Lunch

#### 5.2.1 Heart Rate

**FIGURE 3:**
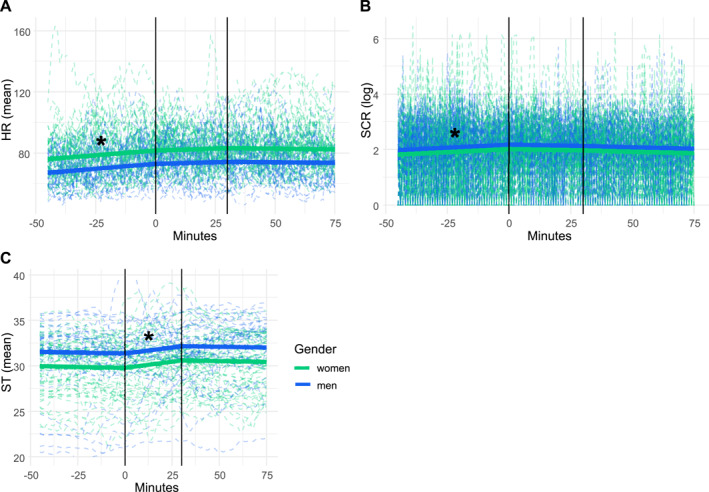
Physiological variables over time relative to meal study 2. The vertical lines represent the start and the end of the lunch. The dashed lines are the individuals' observed values, and the solid lines are the predicted models. The blue lines show the results for the men, and the green lines for the women. Panel A shows heart rate (HR), panel B shows skin conductance response (SCR; log‐transformed), and panel C presents skin temperature (ST).

In Study 2, we found a significant increase in HR before (*β* = 0.13 *t (9793)* = 5.05, *p* = < 0.001) lunch, but no significant effect after (*β* = −0.02, *t (9793)* = −0.41, *p* = 0.678) and during (*β* = 0.05, *t (9793)* = 1.21, *p* = 0.226) lunch (see Figure 3A, Supporting Information S1: table S6). [Corrections added on 11 November 2025, after first online publication: In the preceding sentence, ‘–1.39’ and ‘0.164’ have been corrected to ‘–0.41’ and ‘0.678’, respectively.] Both SD of ACC (*β* = 88.52, *t (9793)* = 80.70, *p* = < 0.001) and gender (*β* = −8.78, *t (25)* = −3.01, *p* = 0.006) were significant predictors in Study 2.

**FIGURE 4:**
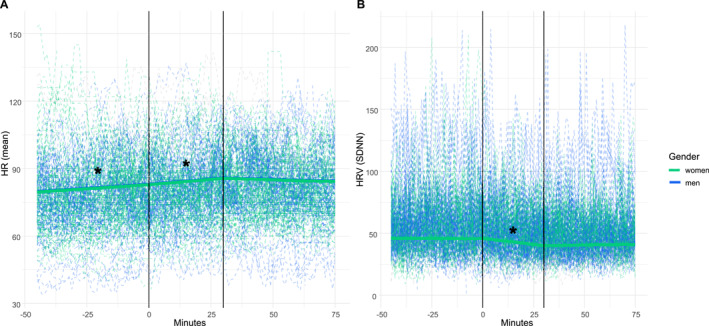
Physiological variables over time relative to meal study 3. The horizontal lines represent the start and the end of the lunch. The blue lines show the results for the men, and the green lines for the women. The dashed lines are the individuals' observed values.

In Study 3, we found a significant increase in HR before lunch (*β* = 0.07, *t* (18,996) = 1.79, *p* = 0.049) and during lunch (*β* = 0.09, *t* (18,996) = 3.03, *p* = 0.002) (Figure 4, Supporting Information S1: Table S7). However, there was no significant effect on HR after lunch (*β* = −0.02, *t* (18,996) = −0.94, *p* = 0.350). Step count was a significant predictor of HR, indicating an increase in HR with increased step coun*t (β* = 0.18, *t (*18,996) = 65.55, *p* < 0.001). However, neither BMI (*β* = 0.01, *t (*18,996) = 0.03, *p* = 0.974) nor gender showed significant effects (male participants: *β* = −1.82, *t (*45) = −0.65, *p* = 0.516).

#### 5.2.2 Heart Rate Variability

In Study 3, we observed a significant decrease in HRV during lunch (*β* = −0.18, *t (*11,675) = −3.92, *p* < 0.001) (Figure 4, Supporting Information S1: Table S7). There was no significant effect on HRV before lunch (*β* = −0.01, *t (*11,675) = −0.26, *p* = 0.795) or after lunch (*β* = 0.01, *t (*11,675) = 0.46, *p* = 0.647). Step count was a significant predictor, indicating a decrease in HRV with increased step count (*β* = −0.10, *t (*11,675) = −15.45, *p* < 0.001). Neither BMI (*β* = 0.21, t (30) = 0.33, *p* = 0.747) nor gender showed significant effects (men: *β* = 3.73, *t (*30) = 0.86, *p* = 0.397).

#### 5.2.3 Skin Conductance Response

In Study 2, we found a significant increase in log SCR before (*β* = 0.00, *t (9076)* = 3.83, *p* = < 0.001) lunch (Figure 3B and Supporting Information S1: Table S6). During lunch (*β* = −0.00, *t (9076)* = −1.06, *p* = 0.291) and after lunch (*β* = −0.00, *t (9076)* = −1.89, *p* = 0.059) there were no significant effects. The standard deviation of ACC (*β* = 3.04, *t (9076)* = 19.68, *p* = < 0.001) was a significant predictor. However, gender (*β* = 0.16, *t (25)* = 1.44, *p* = 0.161) did not predict SCR.

#### 5.2.4 Skin Temperature

In Study 2, there was a significant increase in ST during lunch (*β* = 0.03, *t (11,347)* = 2.87, *p* = 0.004) (Figure 3C and Supporting Information S1: Table S6). We did not find a significant effect before (*β* = −0.00, *t (11,347)* = −0.83, *p* = 0.406) or after (*β* = −0.00, *t (11,347)* = −0.57, *p* = 0.571) lunch. Standard deviation of ACC (*β* = −0.71, *t (11,347)* = −7.17, *p* = < 0.001) was a significant predictor. However, Gender (*β* = 1.58, *t (25)* = 1.53, *p* = 0.138) did not predict ST.

### 5.3 Subjective Stress During Lunch

As expected, we found that HC were, on average, not stressed during lunch in both study samples, with a mean of 44.02 (SD = 16.94; range 3–70) in Study 2 and 37.30 (SD = 15.50; range 12–82) in Study 3 from relaxed (0) to stressed (100).

## 6 General Discussion

The first study investigated physiological responses in individuals with ED during lunch. Contrary to our hypotheses, HR did not show the anticipated increase before the meal nor a decrease afterward. This finding diverges from previous laboratory studies, which reported increased HR in individuals with AN and BN when exposed to food or pictures of high‐calorie food (Gordon et al. 2001; Gorini et al. 2010). However, our analysis showed a decrease in HR during the meal. Contrary to our hypothesis, HR significantly increased after lunch in individuals with BN. This may be due to participants being required to remain in the lunchroom, potentially impeding their ability to engage in compensatory behaviours, which could have elevated stress levels. However, this is a post hoc interpretation that should be considered with caution. Future studies should examine it directly. Similarly, HRV did not exhibit changes across pre‐, during and post‐meal periods.

Furthermore, we found no significant changes in SCR before, during or after the meal, which challenges our assumption that food‐related anxiety would heighten SNS activity. This outcome is consistent with earlier findings of reduced SCR to food cues in individuals with AN compared to HC (Soussignan et al. 2011). Additionally, ST did not show the expected decrease before or increase afterward in individuals with AN. However, we found a pre‐meal decrease in ST, and a post‐meal increase in ST in BN, which might suggest some diagnosis‐specific physiological responses. Despite the lack of significant changes in physiological markers, we observed a notable increase in subjective stress during the meal. This supports the idea that mealtimes are highly stressful for individuals with ED, consistent with prior research (Accurso et al. 2023; Lloyd et al. 2021). The subjective stress levels did not differ significantly between AN and BN.

A possible explanation for the observed discrepancy between objective physiological measures and subjective stress reports may be a dissociation between emotional and physiological stress responses in individuals with ED. Chronic stress or prolonged undernutrition, particularly in individuals with AN, might lead to attenuated physiological reactivity and impaired SNS functioning, resulting in lower HR and SCR despite high levels of self‐reported stress (Jenkins et al. 2021). Although BMI was included as a covariate and did not significantly predict physiological responses in our analyses, the influence of underweight status on autonomic reactivity remains a relevant consideration. This interpretation is supported by previous research showing attenuated HR and HRV responses following a psychosocial stress task in individuals with AN (Schmalbach et al. 2021), as well as by a meta‐analysis revealing reduced SNS activity in AN across various interpersonal stress paradigms, as indicated by blunted HR and lower alpha‐amylase responses (Monteleone et al. 2018). Importantly, such emotional‐physiological dissociations may not be unique to ED but may also occur in other psychiatric conditions, such as borderline personality disorder (Nater et al. 2010), suggesting broader disruptions in stress regulation rather than disorder‐specific patterns.

We included Studies 2 and 3 alongside Study 1 to address a gap in the literature regarding physiological responses to meals in non‐clinical populations using comparable time windows and temporal resolution. These studies were not intended for direct comparison but to serve as contextual, exploratory reference points for interpreting physiological patterns in a real‐world setting. In both Study 2 and Study 3, HR increased before lunch, and in Study 3, HR also increased during lunch. Additionally, HRV (SDNN) decreased during lunch. SCR showed an increase before lunch. Since the HC did not report high levels of subjective stress, these physiological changes might reflect other biological or emotional responses to food or the social aspect of lunchtime. Moreover, office workers often move before lunch (e.g., walking to the canteen), which also results in ANS changes. However, we did include movement as a covariate in the model. The observed increase in HR during the meal may be attributed to an enhanced SNS response. This response acts as a compensatory mechanism for the postprandial reduction in peripheral vascular resistance caused by vasodilation and the subsequent rise in blood flow to the gastrointestinal tract (van Baak 2008). A postprandial increase in HR has been reported both in the short and long term. The short‐term increase is attributed to cephalic and orosensory phase responses to food intake, while the long‐term rise is linked to gastrointestinal mechanisms, both of which result in changes to the ANS response (Harthoorn and Dransfield 2008). Famously first described by Pavlov, cephalic phase responses are anticipatory changes in physiology and metabolism that prepare the digestive tract to digest, absorb, and metabolize food (Power and Schulkin 2008). Moreover, the anticipatory emotional phase may influence ANS activity. A review found that both negative (e.g., anxiety) and positive (e.g., anticipatory pleasure) emotions were associated with increased HR, decreased HRV, and heightened SCR (Kreibig 2010). However, other explanations should be considered, as these specific factors were not directly tested in the current study. ST increased during lunch, which might be attributed to the thermic effect of food following a meal. An increased ST following a test meal has been described previously (Martinez‐Tellez et al. 2019).

There are also several limitations to consider. First, while eating is a well‐documented stressor for individuals with ED, it also naturally influences the ANS in all individuals, regardless of stress. Although we included HC, who did not perceive the meal as stressful, it remains difficult to fully disentangle the effects of eating from those of stress on the autonomic responses observed. Future research could address this by comparing autonomic responses during stress‐inducing tasks that do not involve food with those elicited during mealtimes, to better isolate the physiological impact of eating‐related stress. Second, Study 1 lacks a true baseline, making it difficult to separate mealtime stress from anticipatory arousal. The 45‐min pre‐lunch period likely already includes anticipatory stress in the ED group. Third, given that three different data sets are used, we cannot directly compare individuals with ED and HC. Future studies should aim to include both groups in the same design to allow for a direct analysis of differences in stress reactivity. Fourth, the sample sizes are modest, and Study 1 included only women, limiting the findings' generalisability. Fifth, although all participants in Study 1 were hospitalized for active ED treatment, they varied in treatment duration, illness history, and prior treatment history. Additionally, we included individuals with both typical and atypical AN in a single diagnostic group. Such clinical heterogeneity, typical within ED populations, was not controlled for and may have influenced physiological and subjective responses. Specifically, participants with longer treatment durations might have employed therapeutic strategies, potentially modulating their stress responses during mealtimes. Sixth, in Study 3, HRV was measured using a wrist‐worn wearable, which may offer lower data quality compared to the chest patch used in Study 1. Seventh, the focus on a broader time window around lunch (45 min before to 75 min after the start of the meal) limits our ability to examine second‐by‐second physiological changes during the mealtime itself. Finally, there might be other important variables that we did not assess (e.g., hunger).

One of the key strengths of this study is the comprehensive assessment of various physiological variables, encompassing HR, SDNN, RMSSD, ST, and SCR. Additionally, our study addresses limitations of previous research by investigating the effects of an actual meal in both semi‐controlled (Study 1) and ambulatory (Studies 2 and 3) settings, rather than relying on food cues or imagery typically used in laboratory environments. For individuals with ED, the hospital environment offers the ecological validity associated with a ‘real‐world’ setting while maintaining control over the conditions, given the structured meal schedule. For HC, a smartphone application facilitated self‐reported mealtimes, enhancing the study's relevance to everyday settings. Furthermore, by investigating the trajectories of physiological variables throughout the meal period, rather than relying on isolated pre‐ and post‐meal measurements, we offer a more dynamic and complete picture of the physiological changes that occur.

This study offers potential clinical implications, particularly for using wearable devices as objective markers for monitoring treatment progress. These devices could help detect changes in autonomic regulation during meals, potentially serving as an indicator of recovery in stress resilience (Het et al. 2020). However, it is important to acknowledge the current limitations of wearables in detecting stress in individuals with AN or BN, as their physiological stress markers often do not align with their subjective stress experiences.

## 7 Conclusion

Our findings indicate that while individuals with ED exhibit a significant increase in subjective stress during mealtime, the anticipated physiological stress responses are not observed. This disconnection between physiological stress and subjective stress suggests a possible dysregulation of the ANS in individuals with ED, possibly due to chronic stress or prolonged undernutrition, leading to an attenuated or blunted physiological response. Furthermore, we observed autonomic nervous system changes during lunch in HC, which might indicate biological and emotional responses to lunch unrelated to stress.

## Author Contributions

**Emma De Schuyteneer:** conceptualization, methodology, formal analysis, data curation, visualization, writing – original draft, writing – review and editing. **Robin Quagebeur:** conceptualization, methodology, formal analysis, data curation, visualization, writing – original draft, writing – review and editing. **Femke de Gooijer:** investigation, data curation, review and editing. **Alex van Kraaij:** investigation, data curation, review and editing. **Annelies Goris:** writing – review and editing. **Neide Simões‐Capela:** investigation, data curation, review and editing. **Elske Vrieze:** supervision, writing – review and editing.

## Conflicts of Interest

The authors declare no conflicts of interest.

## Supporting information

Supporting Information S1

## Data Availability

The datasets generated and/or analysed during the first study are available from the corresponding author upon reasonable request. The authors do not have permission to share data from the second and third studies.
