# Supplementary material for: Mealtime Physiological Responses in Individuals With Eating Disorders and Healthy Controls
Source: Eur Eat Disord Rev. 2025 Aug 2;34(1):191–201. doi: 10.1002/erv.70022 (PMC12694697; doi:10.1002/erv.70022)
Supplement: Supplementary file 1 — Supporting Information S1 [file ERV-34-191-s001.docx]

Appendix: supplementary materials

**Background information Study 1**

The data presented in Study 1 were drawn from a larger study investigating physiological and psychological functioning in individuals with ED and HC, using wearable sensors and ecological momentary assessment (EMA). The original study (XXX) addressed two primary research questions: (1) Can individuals with eating disorders be differentiated from healthy controls based on autonomic nervous system signals collected in a naturalistic environment? and (2) Do EMA reports of mood, stress, and contextual factors differ between ED patients and healthy controls?

The full dataset included individuals with a range of DSM-5 ED diagnoses: 26 with anorexia nervosa (AN), 8 with atypical AN, 13 with bulimia nervosa (BN), 5 with binge eating disorder (BED), and 2 with other specified feeding or eating disorders (OSFED). Additionally, 38 healthy controls participated in the broader study.

For the current manuscript, we focused on a subsample of 47 adult women aged 18–50 years (*M* = 26.1, *SD* = 7.4), including those with AN, atypical AN, and BN. Participants with BED and OSFED were excluded due to small group sizes, while healthy controls were excluded because their data lacked time information on mealtime moments, which was needed for the current analysis.

**Lunch meal timing and number of analysed meals per participant (Studies 2 and 3)**

In Study 2, the mean number of lunches included in the analysis per participant was 3.7 (SD = 1.23), with a range of 1 to 5 meals. Reported lunch start times ranged from 11:05 to 15:26.
In Study 3, the mean number of lunches included per participant was 4.74 (SD = 1.25), with a range of 2 to 8 meals. Reported lunch start times ranged from 11:15 to 15:15.

## Pre-processing wearables Study 1

For this paper, we concentrated on physiological data collected with the devices before, during and after lunch between 11:30 am and 1:30 pm. The raw physiological data collected by the wearables were divided into five-minute windows with one-minute overlap (Smets et al., 2018). The ECG R-peaks were detected using an automatic algorithm (Romero et al., 2009). The features were calculated based on the codebook published by Smets et al. (2018). The quality of the ECG features was evaluated in a binary manner based on the study of Orphanidou et al. (2015). For EDA, data quality was assessed according to the study of Boucsein et al. (2012). For skin temperature, data quality was checked according to Jones and Lederman (2006), and only data with a quality score greater than 0.80 were retained. For two participants, the chest patch data was missing. For the Chillband data, the data from the wrist with the least amount of movement was selected and operationalised as the mean of the variable ‘standard deviation ACC’ for each participant separately. Additionally, data with high movement was removed based on the standard deviation of acceleration to avoid the confounding effect of physical activity (Rahman et al., 2014). For the chest patch data, the activity threshold was set at 0.04 m/s^2^ (Smets et al., 2018). For the wristband data, the threshold was set at the 75^th^ percentile of our entire dataset (i.e., 2.7 m/s^2^). Four physiological features were included in our study: two features for ECG, namely mean heart rate and root mean square of successive RR differences (RMSSD); one electrodermal activity feature, skin conductance response peaks per minute (SCR); and one skin temperature feature, mean ST.

## Pre-processing wearables Study 2 and 3

The raw physiological data collected by the Chill+ wristband was pre-processed according to Bögemann et al. (2024). The quality of the EDA data was evaluated based on an in-house developed model-based signal quality indicator (SQI) by IMEC, trained on the annotations of Pattyn et al. (2023). For skin temperature, data quality was checked according to Jones and Lederman (2006).

Eight participants had no Garmin data due to technical issues with the application's synchronisation, resulting in 49 participants for the HR analysis. In addition, only 34 participants had HRV data due to the inability to synchronise the data via iOS phones. The mean heart rate data were extracted directly from Garmin wearable devices, as Beat-to-Beat Interval (BBI) data were not available for all participants. The data were downloaded via the Garmin Connect app. The standard deviation of the NN intervals (SDNN) was calculated from BBI data provided by Garmin.

Table S1

*A sub-analysis Comparing the Anorexia Nervosa (AN) and Bulimia Nervosa (BN) Participants (Study 1)*

|  | **AN (*n* = 34)** | **BN (*n* = 13)** |  |  |
| --- | --- | --- | --- | --- |
|  | *M (SD); range* | *M (SD); range* | test statistic | *p* |
|  |  |  |  |  |
| Age, years | 25.9 (8.4); 18-50 | 26.5 (3.6) 20-34 | *W* = 171 | 0.237 |
| BMI | 17 (2.8); 11.5-21.7 | 22.5 (3.0); 17.8-28.6 | ***t* = -5.71,**  **df = 20.193** | **<0.001** |
| Days since admission | 38.1 (29.4); 3-98 | 32.2 (30.3);3-105 | *W* = 251 | 0.483 |
| EDI total score | 174.0 (49.8); 85-285 | 177.7 (33.6); 107-210 | *W* = 212 | 0.840 |
| EDI chasing thinness | 19.6 (7.8); 1-28 | 19.4 (5.9); 11-28 | *W* = 241.5 | 0.634 |
| EDI bulimia | 5.4 (7.2); 0-29 | 16.2 (7.2); 3-26 | ***W* = 57** | **<0.001** |
| EDI dissatisfaction with body | 28.2 (10.3); 4-40 | 28.5 (7.7); 15-37 | *W* = 229 | 0.858 |
| PSS score | 28.5 (4.0); 20-37 | 26.3 (6.2); 14-36 | *W* = 262.5 | 0.327 |
| DEBQ restrained eating | 4.2 (0.8); 2-5 | 4.0 (0.8); 2.8-5 | *W* = 244.5 | 0.583 |
| DEBQ external eating | 2.5 (0.9); 1-4.4 | 3.0 (0.7); 1.3-4 | *W* = 143 | 0.06 |
| DEBQ emotional eating | 1.9 (1.0); 1-4.8 | 3.4 (0.8); 1.7-4.7 | ***W* = 56** | **<0.001** |
| Treatment program, *n* |  |  |  |  |
| Day hospital | 2 | 1 |  |  |
| Day program | 5 | 3 |  |  |
| Inpatient | 27 | 9 |  |  |
| Medication, *n* |  |  |  |  |
| Antidepressant | 19 | 6 |  |  |
| Antipsychotic | 7 | 1 |  |  |
| Anxiolytic | 7 | 0 |  |  |
| Sedative | 9 | 0 |  |  |

Table S2

|  | **Subjective Stress** | | | | | |
| --- | --- | --- | --- | --- | --- | --- |
| **Predictors** | β | *SE* | *CI* | *t* | *p* | *df* |
| Intercept | 53.01 | 3.68 | 45.73 – 60.28 | 14.41 | **<0.001** | 134 |
| Time [before] | -12.08 | 3.67 | -19.34 – -4.83 | -3.30 | **0.001** | 134 |
| Time [after] | -13.17 | 3.67 | -20.42 – -5.92 | -3.59 | **<0.001** | 134 |
| *n* | 47 | | | | | |
| Observations | 139 | | | | | |
| Marginal *R^2^*/ Conditional *R^2^* | 0.054 / 0.537 | | | | | |

*Linear Mixed Model of Subjective Stress with Time as Predictor*

*Note. Reference category for Time = during lunch*

Table S3

*Linear Mixed Model of Subjective Stress with Time, Diagnosis, and the Interaction as Predictors in Individuals with Eating Disorders (Study 1)*

|  | **Subjective Stress** | | | | | |
| --- | --- | --- | --- | --- | --- | --- |
|  | β | *SE* | *CI* | *t* | *p* |  |
| Fixed effects |  |  |  |  |  |  |
| Intercept | 56.53 | 4.26 | 48.10 – 64.96 | 13.26 | **<0.001** |  |
| Time [before] | -14.00 | 4.31 | -22.52 – -5.48 | -3.25 | **0.001** |  |
| Time [after] | -14.32 | 4.31 | -22.83 – -5.80 | -3.32 | **0.001** |  |
| Diagnosis [BN] | -12.73 | 8.10 | -28.76 – 3.31 | -1.57 | 0.119 |  |
| Time [before] × Diagnosis [BN] | 6.91 | 8.13 | -9.17 – 22.99 | 0.85 | 0.397 |  |
| Time [after] × Diagnosis [BN] | 4.24 | 8.31 | -11.84 – 20.32 | 0.52 | 0.603 |  |
| *n* | 47 | | | | | |
| Observations | 139 | | | | | |
| *R^2^_m_*/ *R^2^_c_* | 0.081 / 0.540 | | | | | |

*Note.* Reference category for Time = during lunch. *R^2^_m_*= marginal R^2^ and *R^2^_c_* conditional R^2^

Table S4

*Piecewise Linear Mixed Model of Physiological Variables in Individuals with Eating Disorders (Study 1)*

|  | **HR (mean)** | | | | **HRV (RMSSD)** | | | | **SCR (log)** | | | | **ST (mean)** | | | |
| --- | --- | --- | --- | --- | --- | --- | --- | --- | --- | --- | --- | --- | --- | --- | --- | --- |
|  | β | CI | t | p | β | CI | t | p | β | CI | t | p | β | CI | t | p |
| Fixed effects | | | | | | | | | | | | | | | | |
| Intercept | 88.91 | 70.14 – 107.67 | 9.28 | **<0.001** | 70.39 | 10.70 – 130.08 | 2.31 | **0.021** | 1.08 | -0.92 – 3.08 | 1.06 | 0.291 | 31.41 | 29.11 – 33.71 | 26.72 | **<0.001** |
| Time [before] | -0.00 | -0.05 – 0.05 | -0.06 | 0.951 | 0.14 | -0.11 – 0.38 | 1.09 | 0.277 | 0.01 | -0.00 – 0.02 | 1.92 | 0.055 | 0.00 | -0.02 – 0.02 | 0.31 | 0.754 |
| Time [during] | -0.13 | -0.19 – -0.07 | -4.04 | **<0.001** | -0.00 | -0.44 – 0.44 | -0.01 | 0.989 | 0.00 | -0.01 – 0.02 | 0.76 | 0.446 | 0.00 | -0.01 – 0.02 | 0.38 | 0.701 |
| Time [after] | 0.05 | -0.01 – 0.10 | 1.58 | 0.114 | 0.14 | -0.21 – 0.49 | 0.76 | 0.446 | -0.01 | -0.02 – 0.00 | -1.28 | 0.199 | -0.00 | -0.02 – 0.01 | -0.61 | 0.542 |
| BMI | -0.51 | -1.51 – 0.49 | -1.02 | 0.312 | -0.80 | -3.97 – 2.37 | -0.51 | 0.613 | 0.04 | -0.07 – 0.14 | 0.69 | 0.497 | 0.02 | -0.10 – 0.14 | 0.33 | 0.741 |
| ACC | 110.84 | 93.12 – 128.56 | 12.25 | **<0.001** | 1071.58 | 932.62 – 1210.54 | 15.10 | **<0.001** | -0.30 | -1.14 – 0.53 | -0.71 | 0.476 | 0.08 | -0.29 – 0.45 | 0.42 | 0.678 |
| *n* | 45 | | | | 45 | | | | 41 | | | | 41 | | | |
| Observations | 2883 | | | | 2883 | | | | 2908 | | | | 2908 | | | |
| *R^2^_m_*/ *R^2^_c_* | 0.029 / 0.962 | | | | 0.029 / 0.813 | | | | 0.018 / 0.739 | | | | 0.002 / 0.963 | | | |

Table S5

*Exploratory Piecewise Linear Mixed Model of Physiological Variables with an Interaction with Diagnosis in Individuals with Eating Disorders (Study 1)*

|  | **HR (mean)** | | | | **HRV (RMSSD)** | | | | | | **SCR (log)** | | | | **ST (mean)** | | | | | | |
| --- | --- | --- | --- | --- | --- | --- | --- | --- | --- | --- | --- | --- | --- | --- | --- | --- | --- | --- | --- | --- | --- |
|  | β | *CI* | *t* | *p* | | β | | *CI* | *t* | *p* | | β | *CI* | *t* | | *p* | β | | *CI* | *t* | *p* |
| Fixed effects | | | | | | | | | | | | | | | | | | | | | |
| (Intercept) | 75.75 | 53.80-97.60 | 6.76 | **<0.001** | | 103.66 | | 32.06 – 175.27 | 2.83 | **0.005** | | 1.57 | -0.81 – 3.96 | 1.29 | | 0.197 | 31.52 | | 28.75 – 34.28 | 22.31 | **<0.001** |
| Time [before] | 0.03 | -0.03 – 0.08 | 0.93 | 0.350 | | 0.20 | | -0.10 – 0.49 | 1.30 | 0.192 | | 0.01 | -0.00 – 0.03 | 1.81 | | 0.070 | 0.01 | | -0.01 – 0.03 | 1.31 | 0.191 |
| Time [during] | -0.14 | -0.22 – -0.07 | -3.76 | **<0.001** | | 0.25 | | -0.25 – 0.75 | 0.97 | 0.330 | | 0.00 | -0.01 – 0.02 | 0.39 | | 0.700 | 0.00 | | -0.01 – 0.02 | 0.43 | 0.667 |
| Time [after] | 0.01 | -0.05 – 0.07 | 0.29 | 0.775 | | 0.05 | | -0.37 – 0.46 | 0.22 | 0.829 | | -0.01 | -0.02 – 0.01 | -0.89 | | 0.374 | -0.01 | | -0.02 – 0.00 | -1.87 | 0.062 |
| Diagnosis [BN] | -16.21 | -27.08 - -5.35 | -3.01 | **0.004** | | 37.66 | | -0.61-75.92 | 1.98 | 0.054 | | 0.24 | -1.06 – 1.53 | 0.37 | | 0.715 | -0.10 | | -1.67 – 1.46 | -0.13 | 0.894 |
| BMI | 0.44 | -0.83 – 1.72 | 0.70 | 0.488 | | -3.16 | | -7.30 – 0.97 | -1.54 | 0.131 | | 0.01 | -0.13 – 0.14 | 0.10 | | 0.923 | 0.02 | | -0.14 – 0.17 | 0.20 | 0.846 |
| ACC | 110.79 | 93.07 – 128.51 | 12.24 | **<0.001** | | 1070.76 | | 931.96 – 1209.57 | 15.10 | **<0.001** | | -0.30 | -1.14 – 0.53 | -0.71 | | 0.475 | 0.08 | | -0.29 – 0.45 | 0.41 | 0.685 |
| Time [before] x  Diagnosis [BN] | -0.09 | -0.19 – 0.01 | -1.79 | 0.074 | | -0.20 | | -0.73 – 0.33 | -0.74 | 0.461 | | -0.01 | -0.03 – 0.02 | -0.34 | | 0.735 | -0.05 | | -0.09 – -0.00 | -2.11 | **0.035** |
| Time [during] x  Diagnosis [BN] | 0.05 | -0.08 – 0.18 | 0.75 | 0.454 | | -0.78 | | -1.68 – 0.12 | -0.69 | 0.091 | | 0.01 | -0.02 – 0.04 | 0.64 | | 0.520 | -0.00 | | -0.04 – 0.03 | -0.16 | 0.870 |
| Time [after] x  Diagnosis [BN] | 0.12 | 0.00 – 0.23 | 1.98 | **0.048** | | 0.30 | | -0.45 – 1.05 | 0.79 | 0.428 | | -0.01 | -0.04 – 0.02 | -0.55 | | 0.585 | 0.04 | | 0.01 – 0.06 | 2.66 | **0.008** |
| *n* | 45 | | | | | | 45 | | | | | 41 | | | | | | 41 | | | |
| Observations | 2883 | | | | | | 2883 | | | | | 2908 | | | | | | 2908 | | | |
| *R^2^_m_*/ *R^2^_c_* | 0.182 / 0.963 | | | | | | 0.087 / 0.814 | | | | | 0.023 / 0.740 | | | | | | 0.041 / 0.963 | | | |

Table S6

*Piecewise Linear Mixed Model of Physiological Variables in HC (Study 2)*

|  | **HR (mean)** | | | | **SCR (log)** | | | | **ST (mean)** | | | |
| --- | --- | --- | --- | --- | --- | --- | --- | --- | --- | --- | --- | --- |
|  | β | *CI* | *t* | *p* | β | *CI* | *t* | *p* | β | *CI* | *t* | *p* |
| Fixed effects |  |  |  |  |  |  |  |  |  |  |  |  |
| (Intercept) | 74.47 | 71.00 – 77.94 | 42.05 | **<0.001** | 1.77 | 1.63 – 1.90 | 25.96 | **<0.001** | 29.83 | 28.57 – 31.10 | 46.20 | **<0.001** |
| Time [before] | 0.13 | 0.08 – 0.18 | 5.05 | **<0.001** | 0.00 | 0.00 – 0.01 | 3.83 | **<0.001** | -0.00 | -0.01 – 0.01 | -0.83 | 0.406 |
| Time [during] | 0.05 | -0.03 – 0.12 | 1.21 | 0.226 | -0.00 | -0.00 – 0.00 | -1.06 | 0.291 | 0.03 | 0.01 – 0.05 | 2.87 | **0.004** |
| Time [after] | -0.02 | -0.09 – 0.06 | -0.41 | 0.678 | -0.00 | -0.00 – -0.00 | -1.89 | 0.059 | -0.00 | -0.02 – 0.01 | -0.57 | 0.571 |
| ACC magnitude std | 88.52 | 86.37 – 90.67 | 80.70 | **<0.001** | 3.04 | 2.74 – 3.35 | 19.68 | **<0.001** | -0.71 | -0.91 – -0.52 | -7.17 | **<0.001** |
| gender [male] | -8.78 | -14.80 – -2.77 | -3.01 | **0.006** | 0.16 | -0.07 – 0.38 | 1.44 | 0.161 | 1.58 | -0.54 – 3.69 | 1.53 | 0.138 |
| *n* | 27 | | | | 27 | | | | 27 | | | |
| Observations | 9895 | | | | 9177 | | | | 11449 | | | |
| *R^2^_m_*/ *R^2^_c_* | 0.102 / 0.930 | | | | 0.034 / 0.493 | | | | 0.004 / 0.997 | | | |

Table S7

*Piecewise Linear Mixed Model of Physiological Variables in HC (Study 3)*

|  | **HR** | | | | **HRV (SDNN)** | | | |
| --- | --- | --- | --- | --- | --- | --- | --- | --- |
|  | β | *CI* | *t* | *p* | β | *CI* | *t* | *p* |
| Fixed effects |  |  |  |  |  |  |  |  |
| Intercept | 78.78 | 60.09 – 97.46 | 8.26 | **<0.001** | 47.72 | 20.62 – 74.81 | 3.45 | **<0.001** |
| Time [before] | 0.07 | 0.00 – 0.14 | 1.79 | **0.049** | -0.01 | -0.10 – 0.08 | -0.26 | 0.795 |
| Time [during] | 0.09 | 0.03 – 0.15 | 3.03 | **0.002** | -0.18 | -0.28 – -0.09 | -3.92 | **<0.001** |
| Time [after] | -0.02 | -0.07 – 0.03 | -0.94 | 0.350 | 0.01 | -0.05 – 0.08 | 0.46 | 0.647 |
| Step count | 0.18 | 0.17 – 0.18 | 65.55 | **<0.001** | -0.10 | -0.12 – -0.09 | -15.45 | **<0.001** |
| BMI | 0.01 | -0.84 – 0.87 | 0.03 | 0.974 | 0.21 | -1.08 – 1.49 | 0.33 | 0.747 |
| Gender [male] | -1.82 | -7.43 – 3.79 | -0.65 | 0.516 | 3.73 | -5.14 – 12.60 | 0.86 | 0.397 |
| *n* | 48 | | | | 33 | | | |
| Observations | 19176 | | | | 11791 | | | |
| *R^2^_m_*/ *R^2^_c_* | 0.025/0.935 | | | | 0.015/0.784 | | | |
